# Unplanned Inpatient Admission After Intended Outpatient Total Extraperitoneal Inguinal Hernia Repair: Precautionary Monitoring or Clinically Relevant Morbidity?

**DOI:** 10.3390/jcm15218279

**Published:** 2026-10-24

**Authors:** Julia Michel, Leonie Wesselmann, Fabian Nimczewski, Krzysztof Nowakowski, Zsolt Madarasz, Jens Hoeppner

**Affiliations:** Department of Surgery, Medical School and University Medical Center OWL, Bielefeld University, Campus Hospital Lippe, Röntgenstr. 18, 32756 Detmold, Germanyjens.hoeppner@uni-bielefeld.de (J.H.)

**Keywords:** inguinal hernia, total extraperitoneal repair, outpatient surgery, unplanned admission, Clavien–Dindo classification

## Abstract

**Background/Objectives:** Unplanned inpatient admission after intended outpatient surgery is commonly regarded as failure of ambulatory management, although the clinical relevance of such admissions remains unclear. This study evaluated the causes, management, and short-term outcomes of unplanned inpatient admission after intended outpatient total extraperitoneal (TEP) inguinal hernia repair. **Methods:** This retrospective single-center study included all patients requiring unplanned inpatient admission after intended outpatient unilateral TEP repair between January 2022 and May 2025. Admission triggers, perioperative management, Clavien–Dindo complication severity, length of stay, need for invasive intervention, and 30-day outcomes were analyzed descriptively. **Results:** Among 153 intended outpatient TEP procedures, 45 patients (29.4%) required unplanned inpatient admission. The most frequent trigger was suspected intraoperative bleeding requiring observation (29/45, 64.4%), followed by technical or anatomical factors (6/45, 13.3%), cardiovascular events (4/45, 8.9%), other medical reasons (3/45, 6.7%), postoperative pain or nausea (2/45, 4.4%), and social or organizational reasons (1/45, 2.2%). Thirty-seven patients (82.2%) had no postoperative complication, six (13.3%) had Clavien–Dindo grade I–II complications, and two (4.4%) had grade III or higher complications requiring invasive intervention. Most patients were discharged on postoperative day 1 (35/45, 77.8%). No patient required blood transfusion, intensive care admission, or 30-day readmission. **Conclusions:** Unplanned inpatient admission occurred in 29.4% of intended outpatient TEP procedures, representing a relatively high admission rate for a planned outpatient pathway. Although most admissions were associated with precautionary monitoring rather than clinically relevant morbidity, invasive intervention was required in a small proportion of patients. The retrospective data do not allow individual admissions to be classified as avoidable; however, the findings suggest potential for pathway optimization through more standardized discharge assessment and formalized admission criteria. Prospective studies are required to determine whether such standardization can reduce unplanned inpatient stays while maintaining patient safety.

## 1. Introduction

Outpatient inguinal hernia repair is increasingly used in appropriately selected patients, and total extraperitoneal (TEP) repair represents an established laparo-endoscopic technique for inguinal hernia repair [1,2,3]. An important outcome of outpatient surgical pathways is successful same-day discharge. Consequently, unplanned inpatient admission is frequently interpreted as failure of outpatient management [4,5].

However, unplanned admission does not necessarily reflect clinically relevant postoperative morbidity. Admission may also result from precautionary monitoring after intraoperative concerns, transient postoperative symptoms, technical difficulties, or organizational factors. Studies of ambulatory surgery commonly report unplanned admission rates, but the subsequent clinical course and therapeutic consequences of these admissions are less frequently characterized [4,5]. Distinguishing precautionary observation from admission associated with clinically relevant morbidity may therefore provide a more meaningful assessment of outpatient surgical pathways.

In a previously published institutional cohort of 153 intended outpatient unilateral TEP procedures, 108 patients were discharged on the day of surgery, whereas 45 patients required unplanned inpatient admission [6]. The present study represents a focused secondary analysis of these 45 patients and examines information not addressed in detail in the source publication, including admission triggers, complication severity, therapeutic consequences, length of stay, and short-term outcomes.

The aim of this study was to determine whether unplanned inpatient admissions following intended outpatient TEP repair predominantly represented precautionary monitoring or were associated with clinically relevant morbidity.

## 2. Materials and Methods

### 2.1. Study Design and Patient Selection

This retrospective single-center study included all patients who underwent unplanned inpatient admission following intended outpatient TEP inguinal hernia repair between January 2022 and May 2025 at University Medical Center OWL—Campus Hospital Lippe. All consecutive patients meeting these criteria were included. Patients undergoing planned inpatient procedures were excluded.

This study represents a secondary analysis of a previously published institutional cohort evaluating outpatient TEP repair after implementation of an outpatient surgery pathway [6]. This secondary analysis differs from the previously reported source cohort by focusing exclusively on the subgroup of patients requiring unplanned inpatient admission. The present manuscript does not reassess outpatient feasibility, patient satisfaction, or perceived safety, but specifically evaluates whether unplanned admission represented precautionary monitoring or clinically relevant morbidity.

Admission triggers were categorized according to the predefined categories used in the previously published source cohort to ensure consistency of reporting. In addition, the present analysis assessed the clinical relevance of each admission by evaluating therapeutic consequences, complication severity, length of stay, need for intervention, and 30-day readmission.

Clinically relevant complications were defined as events requiring invasive intervention, corresponding to Clavien–Dindo grade III or higher (CDC ≥ III) [7]. Minor complications were classified separately as Clavien–Dindo grade I–II. Admissions without postoperative complications or therapeutic consequences were classified as precautionary admissions.

### 2.2. Surgical Procedure and Perioperative Management

All procedures were initiated using a standardized TEP technique. Procedures were generally performed by a surgical resident as the primary operator under direct supervision of an experienced senior surgeon. In privately insured patients, the senior surgeon generally served as the primary operator. The supervising senior surgeons had extensive experience in TEP inguinal hernia repair. Surgical drains were placed selectively, particularly in cases of diffuse intraoperative oozing, extensive hemostasis, technically difficult dissection, or a large dissected preperitoneal space. In the absence of these intraoperative concerns, a drain was not routinely placed. Drain placement alone was not considered an indication for inpatient admission. Patients who otherwise fulfilled the discharge criteria could be discharged with the drain in situ and were routinely reassessed on the following day for clinical evaluation and drain removal, when appropriate.

No standardized discharge score, such as the Aldrete score or Post-Anesthetic Discharge Scoring System (PADSS), was routinely used. The decision regarding same-day discharge was made by the operating surgeon after postoperative reassessment of the patient. Patients were considered suitable for same-day discharge if they were hemodynamically stable, awake and oriented, sufficiently mobile, had adequate pain control, no relevant nausea or vomiting, no clinical signs of significant postoperative bleeding, were able to tolerate oral intake, and had voided spontaneously. In cases in which intraoperative concerns had arisen, particularly increased bleeding tendency or the need for extensive hemostasis, the decision regarding postoperative observation and inpatient admission was sometimes made jointly with the supervising senior surgeon. Patients were routinely scheduled for clinical reassessment on the following day, particularly when a surgical drain had been placed, which was generally removed at this visit when appropriate. Patients were instructed to seek medical attention in case of bleeding, increasing swelling, severe pain, fever, nausea or vomiting, and to present to the emergency department in case of acute problems. Further follow-up was provided through our outpatient service and subsequently by the general practitioner as appropriate.

### 2.3. Data Collection and Follow-Up

Clinical and perioperative data were extracted from electronic medical records, including patient characteristics, operative details, reasons for unplanned inpatient admission, postoperative course, and complications.

Short-term follow-up was assessed through review of the hospital information system, outpatient clinic records, emergency presentations, and documented postoperative visits within 30 days.

Systematic 30-day outcome assessment and Clavien–Dindo classification were performed for the unplanned inpatient admission cohort. For the 108 patients discharged on the day of surgery, the previously collected source dataset included perioperative complications but did not provide a complete standardized 30-day Clavien–Dindo classification or systematic 30-day outcome assessment for all patients. Therefore, direct comparison of 30-day morbidity between the two groups was not performed.

### 2.4. Outcome Measures and Statistical Analysis

The primary outcome was clinically relevant morbidity among patients requiring unplanned inpatient admission, defined as a complication requiring invasive intervention and corresponding to Clavien–Dindo grade III or higher (CDC ≥ III). An invasive intervention was defined as any unplanned invasive therapeutic procedure required to treat a postoperative complication. Emergency reoperation was defined as an unplanned return to the operating room for surgical treatment under general anesthesia. Accordingly, ambulatory wound opening and irrigation without general anesthesia was classified as an invasive intervention (CDC IIIa), but not as an emergency reoperation. Additional safety outcomes included blood transfusion, intensive care unit admission, emergency reoperation, and 30-day readmission. Secondary outcomes included admission triggers, length of hospital stay, discharge timing, and postoperative evaluation after discharge.

For contextual purposes, baseline and perioperative characteristics of patients requiring unplanned inpatient admission were descriptively compared with those of patients discharged as planned; this comparison is provided as Supplementary Table S1. Descriptive statistics were used to summarize the data. Continuous variables are presented as mean ± standard deviation or median with interquartile range (IQR) and range, as appropriate, and categorical variables as frequencies and percentages. Distribution of continuous variables was assessed by visual inspection. Given the exploratory descriptive design and the study’s primary focus on the admitted subgroup, no inferential statistical testing was performed.

## 3. Results

**Figure 1:**
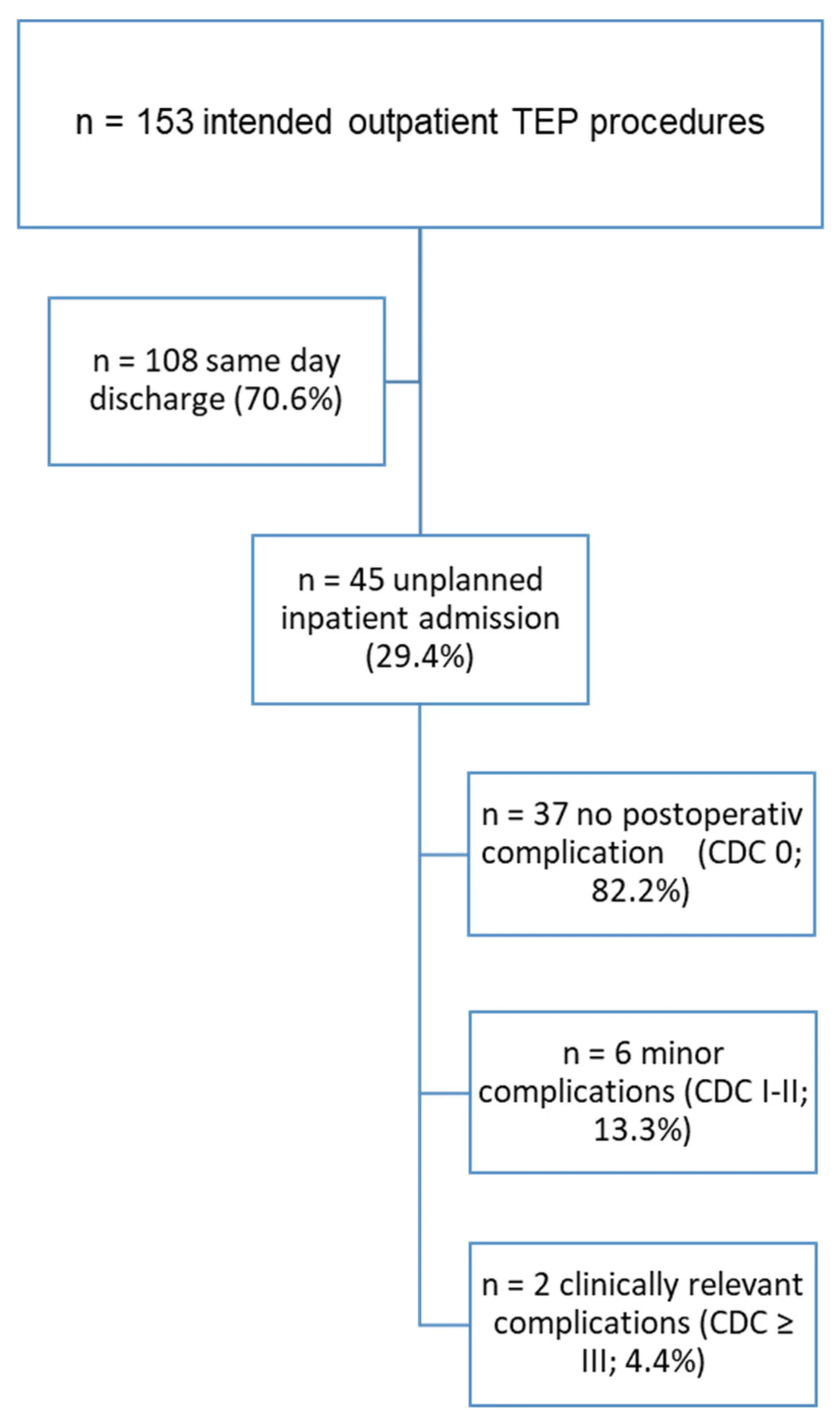
Study flow diagram of intended outpatient TEP procedures and clinical outcome after unplanned inpatient admission.

During the study period, 153 unilateral TEP repairs were scheduled as intended outpatient procedures. Of these, 108 patients were discharged as planned on the day of surgery, whereas 45 patients required unplanned inpatient admission and constituted the study cohort of the present analysis. The overall unplanned inpatient admission rate was 29.4% (Figure 1).

### 3.1. Patients and Procedures

The cohort was predominantly male with a low comorbidity burden (ASA I–II in 82.2%). Operative time and perioperative management parameters are summarized in Table 1.

A descriptive comparison with patients discharged as planned after outpatient TEP repair is provided in Supplementary Table S1. Compared with patients discharged on the day of surgery, patients requiring unplanned inpatient admission had a higher proportion of ASA III status, longer operative time, more frequent drain placement, and a higher rate of surgical conversion to TAPP or Lichtenstein repair. These differences were interpreted descriptively only and were not subjected to inferential testing.

As previously reported for the 108 patients discharged on the day of surgery, seven perioperative complications were documented, comprising four intraoperative bleeding-related events and three postoperative complications (one conservatively managed hematoma and two early recurrences subsequently treated by open mesh repair) [6]. No life-threatening bleeding, emergency reoperation, or intensive care unit admission occurred in this cohort. However, a standardized 30-day Clavien–Dindo classification and complete systematic 30-day outcome assessment were not available for all 108 patients; therefore, no direct comparison of 30-day morbidity with the admitted cohort was performed.

### 3.2. Operative Results

**Figure 2:**
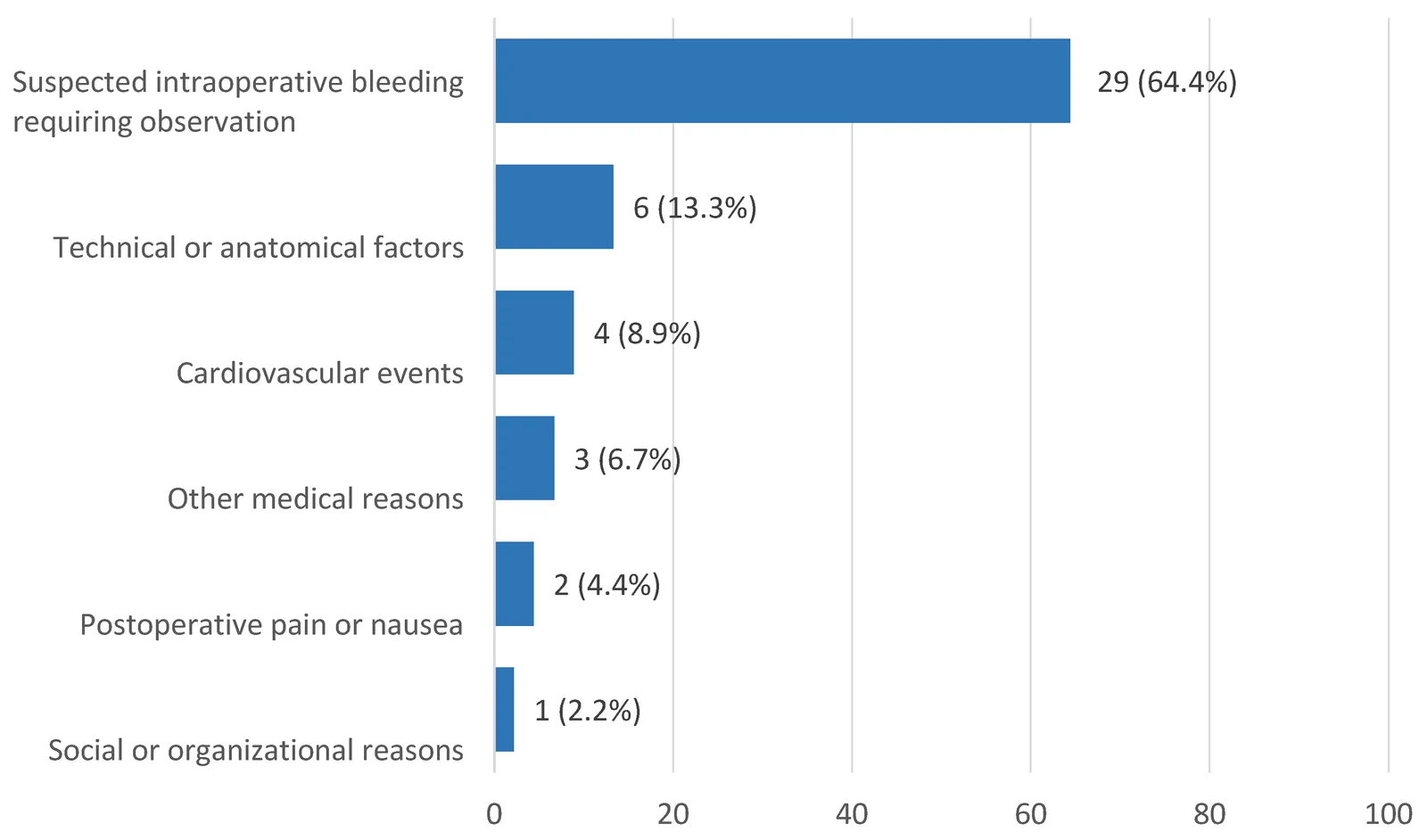
Admission triggers for unplanned inpatient admission after intended outpatient TEP inguinal hernia repair (*n* = 45).

Unplanned inpatient admission occurred in 45 of 153 patients following intended outpatient TEP repair, corresponding to an admission rate of 29.4%. The most frequent admission trigger was suspected intraoperative bleeding requiring postoperative observation, accounting for 64.4% (29/45) of cases. Technical or anatomical factors were responsible for 13.3% (6/45), followed by cardiovascular events in 8.9% (4/45). Other medical reasons accounted for 6.7% (3/45), postoperative pain or nausea for 4.4% (2/45), and social or organizational reasons for 2.2% (1/45) of cases (Figure 2).

Antithrombotic therapy was present in 11.1% (5/45) of patients. Four patients were receiving direct oral anticoagulants (three apixaban and one edoxaban), which were interrupted perioperatively for 48 h, while one patient was receiving clopidogrel, which was continued. Neither of the two patients requiring invasive intervention was receiving antithrombotic therapy.

Intraoperative conversion to an alternative surgical approach was required in 15.6% (7/45) of cases. Conversion from TEP to transabdominal preperitoneal (TAPP) repair occurred in 13.3% (6/45), while one patient (2.2%) required conversion to an open Lichtenstein repair. The majority of procedures (84.4%) were completed as planned using the TEP technique.

Perioperative management data, including drainage placement and tranexamic acid use, are summarized in Table 1. Although a surgical drain was placed in the majority of patients, drainage volumes were generally low, with most patients showing minimal output (<50 mL), and only a small subset exceeding 100 mL. Higher drainage volumes were observed only in a small subset of patients. Bleeding- and technical-related admissions were predominantly triggered intraoperatively, whereas social factors and postoperative pain or nausea were mainly identified postoperatively.

Most admissions were triggered intraoperatively (31/45, 68.9%) rather than postoperatively (14/45, 31.1%). Admission triggers, timing, and clinical relevance are summarized in Table 2.

Overall, 37 patients (82.2%) had no postoperative complications (CDC 0), while 6 patients (13.3%) experienced minor complications (CDC I–II) requiring only conservative management. Clinically relevant complications requiring invasive intervention occurred in two admitted patients (2/45, 4.4%; CDC ≥ III) (Table 2 and Table 3). One patient developed an extensive postoperative paraumbilical subcutaneous hematoma requiring same-day surgical reintervention under general anesthesia, including hematoma evacuation, irrigation, fascial closure, and drain placement (CDC IIIb). A second patient presented on postoperative day 5 with a wound-healing complication requiring ambulatory wound opening and irrigation in the emergency department without general anesthesia (CDC IIIa). No patient required intensive care unit admission or blood transfusion.

Length of stay was short, with a median hospital stay of 1 day. Most patients (35/45, 77.8%) were discharged on the first postoperative day, while only 17.8% required a 2-day stay and 4.4% remained hospitalized for 3 days. Prolonged hospitalization was mainly associated with transient postoperative symptoms such as pain or dizziness rather than complications requiring invasive intervention.

No 30-day readmissions were observed. Three patients (3/45, 6.7%) presented for postoperative evaluation after discharge. Two were managed conservatively for minor symptoms, whereas one patient presented on postoperative day 5 with a wound-healing complication requiring ambulatory wound opening and irrigation in the emergency department (CDC IIIa). No delayed bleeding after discharge or hernia recurrence was observed during follow-up.

## 4. Discussion

The present study provides a focused analysis of unplanned inpatient admissions following intended outpatient TEP inguinal hernia repair, with particular emphasis on their clinical relevance. The main finding is that most admissions were precautionary in nature, whereas clinically relevant complications requiring invasive intervention were rare. Although the overall unplanned inpatient admission rate was 29.4%, clinically relevant complications requiring invasive intervention occurred in only two of the 45 patients requiring unplanned inpatient admission (4.4%). No patient required blood transfusion, intensive care unit admission, or 30-day readmission. The two clinically relevant complications consisted of one postoperative hematoma requiring same-day surgical reintervention under general anesthesia (CDC IIIb) and one wound-healing complication requiring ambulatory wound opening and irrigation on postoperative day 5 (CDC IIIa). Importantly, the latter was managed in the emergency department without subsequent inpatient readmission.

This analysis expands on previously published aggregate data from our institution by examining the clinical course and therapeutic consequences of unplanned inpatient admission in greater detail. While admission triggers are reported consistently with the previously published cohort, the present study adds a clinically oriented assessment of complication severity, inpatient management, length of stay, need for intervention, and short-term outcomes. This distinction is clinically relevant because the implications of unplanned admission may vary substantially.

The unplanned admission rate of 29.4% observed in the present cohort was relatively high compared with previously published day-surgery series. Steger et al. [2] reported unplanned inpatient admission in 12 of 164 patients (7.3%) scheduled for outpatient unilateral TEP repair, mainly because of circulatory disturbances, pain, or bleeding. In a series of 915 patients scheduled for day-case laparoscopic inguinal hernia repair, 93 patients (10.2%) required an unexpected overnight stay; older age, longer operative time, and later position on the operating list were identified as associated factors [8]. In contrast, Wakasugi et al. [9] reported same-day discharge in all 2148 patients treated with single-incision TEP at a specialized day-surgery clinic. Differences in patient selection, surgical and anesthetic techniques, institutional experience, postoperative monitoring, and admission thresholds limit direct comparison between these studies. Nevertheless, the comparatively high admission rate in our cohort suggests that institutional practice and a low threshold for precautionary observation may have contributed substantially to the observed rate. Suspected intraoperative bleeding requiring observation was the most frequent admission trigger, accounting for 29 of 45 admissions (64.4%). This finding reflects one of the key concerns associated with outpatient laparo-endoscopic inguinal hernia repair, namely the potential risk of occult or delayed bleeding in the preperitoneal space. However, postoperative findings did not indicate clinically significant hemorrhage in most patients. Drain output was generally low, no patient required blood transfusion or intensive care, and invasive intervention was required only in a small minority of cases. These findings are consistent with registry-based analyses demonstrating low rates of clinically relevant postoperative bleeding and rare need for reintervention after laparo-endoscopic inguinal hernia repair [10]. Postoperative bleeding risk is multifactorial. Registry data have identified higher age, higher ASA classification, recurrent hernia, larger hernia defect size, male sex, and open surgical repair as additional factors associated with postoperative bleeding, independent of antithrombotic therapy or coagulopathy [10]. Therefore, antithrombotic therapy should not be interpreted as an isolated determinant of bleeding risk in this cohort.

The high proportion of drain placement among admitted patients should be interpreted in the context of the clinical situations that prompted drain use. Drains were preferentially placed in cases of diffuse oozing, extensive hemostasis, technically difficult dissection, or extensive preperitoneal dissection. Thus, drain placement likely represents a marker of intraoperative concern rather than an independent reason for admission. Importantly, drain placement alone did not mandate inpatient observation, as patients fulfilling the clinical discharge criteria could be discharged with the drain in situ and return the following day for reassessment and removal.

Technical or anatomical factors accounted for a smaller proportion of admissions and were mainly related to difficult dissection, adhesions, or challenges in establishing the preperitoneal working space. Intraoperative conversion to an alternative surgical approach was required in a minority of patients. Importantly, these cases were not associated with a relevant increase in postoperative morbidity, suggesting that unplanned admission may represent a reasonable safety measure in technically demanding situations.

Cardiovascular events, including bradycardia and syncope, were infrequent and transient. Postoperative pain or nausea accounted for only two admissions (4.4%), and social or organizational reasons for only one admission (2.2%). Although uncommon, these non-surgical triggers illustrate that real-world outpatient pathways are influenced not only by operative findings, but also by postoperative symptoms, patient perception, discharge logistics, and availability of home support. Similar factors have been described as contributors to unplanned admission after ambulatory surgery in broader surgical cohorts [11,12,13]. The short length of stay further supports the interpretation that most unplanned admissions served as temporary observation rather than prolonged inpatient treatment. More than three quarters of patients were discharged on the first postoperative day, and prolonged hospitalization was mainly associated with transient symptoms rather than complications requiring invasive intervention. No delayed bleeding after discharge, hernia recurrence, or 30-day readmission was observed during short-term follow-up.

Taken together, the unplanned inpatient admission rate of 29.4% represents a substantial proportion of patients initially scheduled for outpatient TEP repair. Although most admitted patients did not subsequently develop clinically relevant morbidity requiring invasive intervention, the retrospective design does not allow individual admissions to be classified reliably as necessary or avoidable. The predominance of precautionary monitoring, the absence of a standardized discharge scoring system, and the discretionary nature of admission decisions suggest that a low institutional threshold for inpatient observation may have contributed to the admission rate. More standardized discharge assessment and formalized admission criteria may therefore help reduce potentially avoidable inpatient stays while maintaining appropriate patient safety.

This study has several limitations. First, it is a retrospective single-center analysis with a relatively small sample size, limiting generalizability. Second, the present study focused primarily on patients requiring unplanned inpatient admission. Although a descriptive comparison with patients discharged as planned is provided, no inferential testing or multivariable risk factor analysis was performed; therefore, no conclusions regarding independent predictors of unplanned admission can be drawn. Surgeon-specific case volumes and outcomes were not analyzed separately; therefore, a potential influence of individual operator experience on operative time or admission decisions cannot be assessed from the present data. Furthermore, although perioperative complications in the same-day discharge cohort were documented in the source study, systematic 30-day Clavien–Dindo classification and complete standardized 30-day surveillance were not available for all 108 patients, precluding a direct comparison of 30-day morbidity between the groups. Third, the decision for inpatient admission was based on surgeon discretion and was not guided by a standardized scoring system or formalized admission criteria, which may have introduced selection and observer bias. Fourth, the classification of admission triggers was based on retrospective documentation and may therefore be subject to information bias. Fifth, follow-up was limited to short-term clinical outcomes recorded in the hospital information system and outpatient records. Finally, the findings reflect the implementation experience of a single institution and may depend on local infrastructure, discharge thresholds, and postoperative follow-up pathways.

## 5. Conclusions

Unplanned inpatient admission occurred in 29.4% of intended outpatient TEP procedures, representing a relatively high admission rate for a planned outpatient pathway. Although most admissions were associated with precautionary monitoring rather than clinically relevant morbidity, invasive intervention was required in a small proportion of patients. The retrospective data do not allow individual admissions to be classified as avoidable; however, the findings suggest potential for pathway optimization through more standardized discharge assessment and formalized admission criteria. Prospective studies are required to determine whether such standardization can reduce unplanned inpatient stays while maintaining patient safety.

## Figures and Tables

**Table 1:** Baseline, hernia, and operative characteristics of patients requiring unplanned inpatient admission (*n* = 45).

Variable	Value (Mean ± SD or *n* [%])
Demographics	
Male sex, *n* (%)	38 (84.4)
Female sex, *n* (%)	7 (15.6)
Age, years, mean ± SD	58.7 ± 14.8
Body mass index, kg/m^2^, mean ± SD	25.8 ± 3.6
Clinical characteristics	
ASA I, *n* (%)	10 (22.2)
ASA II, *n* (%)	27 (60.0)
ASA III, *n* (%)	8 (17.8)
Hernia characteristics	
Lateral hernia, *n* (%)	31 (68.9)
Medial hernia, *n* (%)	8 (17.8)
Combined hernia, *n* (%)	6 (13.3)
Operative characteristics	
Operative time, min, mean ± SD	48.2 ± 20.2
Antithrombotic therapy, *n* (%)	5 (11.1)
Perioperative management	
Drain placement, *n* (%)	34 (75.6)
Tranexamic acid, *n* (%)	3 (6.7)
Surgical strategy	
TEP completed, *n* (%)	38 (84.4)
Conversion to TAPP, *n* (%)	6 (13.3)
Conversion to Lichtenstein repair, *n* (%)	1 (2.2)

TAPP, transabdominal preperitoneal.

**Table 2:** Admission triggers, timing, and clinical relevance of unplanned inpatient admission (*n* = 45).

Admission Trigger	*n* (%)	Intraoperative Trigger	Postoperative Trigger	CDC 0 ^a^	CDC I–II ^a^	CDC ≥ III ^a^	Intervention
Suspected intraoperative bleeding requiring observation	29 (64.4)	23	6	27	0	2	2
Technical or anatomical factors	6 (13.3)	5	1	6	0	0	0
Cardiovascular events	4 (8.9)	1	3	1	3	0	0
Other medical reasons	3 (6.7)	2	1	2	1	0	0
Postoperative pain or nausea	2 (4.4)	0	2	0	2	0	0
Social or organizational reasons	1 (2.2)	0	1	1	0	0	0
Total	45 (100)	31	14	37	6	2	2

^a^ CDC grade reflects the highest postoperative complication within 30 days among patients grouped according to their initial admission trigger; complications were not necessarily causally related to the admission trigger.

**Table 3:** Hospital course and short-term outcomes after unplanned inpatient admission (*n* = 45).

Outcome	Value
Length of stay, days, median (IQR; range)	1 (1–2; 1–3)
Discharge on POD1, *n* (%)	35 (77.8)
Hospital stay 2 days, *n* (%)	8 (17.8)
Hospital stay 3 days, *n* (%)	2 (4.4)
No postoperative complication (CDC 0), *n* (%)	37 (82.2)
Minor complication, CDC I–II, *n* (%)	6 (13.3)
Clinically relevant complication, CDC ≥ III, *n* (%)	2 (4.4)
Blood transfusion, *n* (%)	0
ICU admission, *n* (%)	0
^a^ Emergency reoperation, *n* (%)	1 (2.2)
30-day readmission, *n* (%)	0
Post-discharge evaluation, *n* (%)	3 (6.7)
Delayed bleeding after discharge, *n* (%)	0
Hernia recurrence during short-term follow-up, *n* (%)	0

^a^ Emergency reoperation was defined as an unplanned return to the operating room under general anesthesia; ambulatory wound opening and irrigation without general anesthesia was classified as an invasive intervention but not as emergency reoperation.

## Data Availability

The data presented in this study are available upon request from the corresponding author due to privacy and ethical restrictions.

## Supplementary Materials

The following supporting information can be downloaded at: https://www.mdpi.com/article/10.3390/jcm15218279/s1, Table S1: Descriptive comparison of same-day discharge and unplanned inpatient admission cohorts.

## Abbreviations

TEP	Totally extraperitoneal
TAPP	Transabdominal preperitoneal
POD	Postoperative day
ASA	American Society of Anesthesiologists
SD	Standard deviation
CDC	Clavien–Dindo classification

## References

[1] The HerniaSurge Group (2018). International guidelines for groin hernia management. Hernia.

[2] Steger U., Bisping M., Urban J., Vowinkel T., Wiesmann A., Ryll J. (2019). Minimalinvasive Leistenhernienchirurgie—TEP ambulant. Zentralbl. Chir..

[3] Kallianpur A.A., Parshad R., Dehran M., Hazrah P. (2007). Ambulatory total extraperitoneal inguinal hernia repair: Feasibility and impact on quality of life. J. Soc. Laparoendosc. Surg..

[4] Melton M.S., Li Y.J., Pollard R., Chen Z., Hunting J., Hopkins T., Buhrman W., Taicher B., Aronson S., Stafford-Smith M. (2021). Unplanned hospital admission after ambulatory surgery: A retrospective, single cohort study. Can. J. Anaesth..

[5] Köckerling F., Lorenz R., Reinpold W., Zarras K., Conze J., Kuthe A., Lammers B., Stechemesser B., Mayer F., Fortelny R. (2022). What is the reality in outpatient vs inpatient groin hernia repair? An analysis from the Herniamed Registry. Hernia.

[6] Michel J., Nowakowski K., Nimczewski F., Hoeppner J., Madarasz Z. (2026). Outpatient total extraperitoneal (TEP) for inguinal hernia repair following federal German outpatient operation procedure reform: A preliminary single-center descriptive study of same-day discharge, unplanned admission, and patient-reported outcomes. BMC Surg..

[7] Dindo D., Demartines N., Clavien P.A. (2004). Classification of surgical complications: A new proposal with evaluation in a cohort of 6336 patients and results of a survey. Ann. Surg..

[8] Solodkyy A., Feretis M., Fedotovs A., Di Franco F., Gergely S., Harris A.M. (2018). Elective “True Day Case” Laparoscopic Inguinal Hernia Repair in a District General Hospital: Lessons Learned from 1000 Consecutive Cases. Minim. Invasive Surg..

[9] Wakasugi M., Hasegawa J., Ikeda Y. (2021). Single-incision laparoscopic totally extraperitoneal inguinal hernia repair with tumescent local anesthesia: Report of more than 2000 procedures at a day-surgery clinic. Surg. Today.

[10] Köckerling F., Roessing C., Adolf D., Schug-Pass C., Jacob D. (2016). Has endoscopic (TEP, TAPP) or open inguinal hernia repair a higher risk of bleeding in patients with coagulopathy or antithrombotic therapy? Data from the Herniamed Registry. Surg. Endosc..

[11] Tham C., Koh K.F. (2002). Unanticipated admission after day surgery. Singap. Med. J..

[12] Kent C., Metzner J., Bollag L. (2014). An analysis of risk factors and adverse events in ambulatory surgery. Ambul. Anesth..

[13] Bongiovanni T., Parzynski C., Ranasinghe I., Steinman M.A., Ross J.S. (2021). Unplanned hospital visits after ambulatory surgical care. PLoS ONE.

