# Supplementary material for: Unplanned Inpatient Admission After Intended Outpatient Total Extraperitoneal Inguinal Hernia Repair: Precautionary Monitoring or Clinically Relevant Morbidity?
Source: J Clin Med. 2026 Oct 24;15(21):8279. doi: 10.3390/jcm15218279 (PMC13764479; doi:10.3390/jcm15218279)
Supplement: Supplementary file 1 [file jcm-15-08279-s001.zip › jcm-4581818-supplementary.pdf]

Variable	Same-day discharge n = 108	Unplanned admission n = 45
Age, years, mean ± SD	55.9 ± 12.3	58.7 ± 14.8
Male sex, n (%)	102 (94.4)	38 (84.4)
Female sex, n (%)	6 (5.6)	7 (15.6)
BMI, kg/m², mean ± SD	25.2 ± 3.8	25.8 ± 3.6
ASA I, n (%)	39 (36.1)	10 (22.2)
ASA II, n (%)	63 (58.3)	27 (60.0)
ASA III, n (%)	6 (5.6)	8 (17.8)
Lateral hernia, n (%)	66 (61.1)	31 (68.9)
Medial hernia, n (%)	18 (16.7)	8 (17.8)
Combined hernia, n (%)	24 (22.2)	6 (13.3)
Operative time, min, mean ± SD	39.7 ± 11.1	48.2 ± 20.2
Antithrombotic therapy, n (%)	Not systematically recorded*	5 (11.1)
Drain placement, n (%)	6 (5.6)	34 (75.6)
Tranexamic acid, n (%)	Not systematically recorded†	3 (6.7)
TEP completed, n (%)	108 (100.0)	38 (84.4)
Conversion to TAPP, n (%)	0 (0.0)	6 (13.3)
Conversion to Lichtenstein repair, n (%)	0 (0.0)	1 (2.2)

Supplementary Table S1. Descriptive comparison of same-day discharge and unplanned inpatient admission cohorts.

*Antithrombotic therapy was not systematically recorded as a separate variable in the source dataset for the same-day discharge cohort.

†Tranexamic acid use was not systematically recorded as a separate variable in the source dataset for the same-day discharge cohort.

Abbreviations: ASA, American Society of Anesthesiologists; BMI, body mass index; TEP, total extraperitoneal; TAPP, transabdominal preperitoneal.
